# Arrest of Convulsions by the Sinistro-Lateral Posture

**Published:** 1876-08

**Authors:** 


Arrest of Convulsions by the Sinistro-Lateral Posture.—Dr. F. J. Brown states (*The Practitioner*, April, 1876,) that he has seen two cases of convulsions arrested almost instantly by turning the patient over on the left side, a procedure which he adopted from experience of its good effects both during chloroform inhalation and subsequently in the stage of recovery from the anæsthetic, as first employed by Mr. Bader.

Dr. Brown states that "a few months since a man suffering from Bright's disease was seized with uræmic convulsions in my presence. I turned him upon his left side, and the convulsions ceased instantly.

"Recently a man, aged 56 years, in impaired health from chronic catarrh, was seized with unilateral (right) convulsions. His consciousness and power of speech were intact. He had been convulsed for ten minutes when I entered the house, and he was growing worse. I turned him over upon his left side, and the convulsions ceased in about ten or fifteen seconds. He had experienced a similar seizure on December 9, 1875.

"I hasten to report these cases, for I am certain that marvellous results will be obtained in convulsive diseases (possibly even in epilepsy) by sinistro-lateral posture."—*Am. Jour. Med. Sciences.*

